# *PLOS Pathogens* 2014 Reviewer Thank You

**DOI:** 10.1371/journal.ppat.1004748

**Published:** 2015-02-27

The Public Library of Science (PLOS) and the *PLOS Pathogens* editorial team would like to express our tremendous gratitude to all those individuals who participated in the peer review process this past year at *PLOS Pathogens*. As a journal that is run by and for the scientific community, we depend upon you, the community, to communicate our best work. 2014 was great year for *PLOS Pathogens;* during the year, 2,900 reviewers from around the world helped evaluate over 1,200 articles.

The names of our 2014 reviewers are listed in S1 Reviewer List. Thank you to all our reviewers for sharing your expertise with the *PLOS Pathogens* authors. Your efforts are an important part of making the journal a highly read, highly cited, highly regarded open access resource created by and for the benefit of the scientific community.

## Supporting Information

S1 Reviewer List Alphabetical list of 2014 *PLOS Pathogens* reviewers. (PDF)
