# Supplementary material for: PLOS Pathogens 2014 Reviewer Thank You
Source: PLoS Pathog. 2015 Feb 27;11(2):e1004748. doi: 10.1371/journal.ppat.1004748 (PMC4344192; doi:10.1371/journal.ppat.1004748)
Supplement: S1 Reviewer List — (PDF) [file ppat.1004748.s001.pdf]

PLOS | PATHOGENS

PLOS Pathogens would like to thank all those who reviewed on behalf of the journal in 2014:

Pierre Abad
Derek Abbott
Kristina Abel
Ernesto Abel-Santos
Laurent Abi-Rached
Soman Abraham-
Maria Abreu
Hans Ackerman
Margaret Ackerman
Shelley Adamo
Erin Adams
Zach Adelman
Joshua Adkins
Heiko Adler
Toni Aebischer
Martin Aepfelbacher
Mavis Agbandje-McKenna
Vadim Agol
Hector Aguilar
Paul Ahlquist
Rafi Ahmed
Jin-Hyun Ahn
Tero Ahola
Gillian Air
Darrin Akins
Ramesh Akkina
Randy Albrecht
Antonio Alcami
John Alcorn
Anna Aldovini
Courtney Aldrich
Phillip Aldridge
Grace Aldrovandi
James Alexander
James Alfano
Samuel Alizon
Martin Allday
Lee-Ann Allen
Igor Almeida
Scott Alper
Luke Alphey
James Alspaugh
Galit Alter
Marcus Altfeld
Christian Althaus
John Altman
Daniel Altmann
Neal Alto
Kishore Alugupalli
Jose Alves-Filho
James Alwine
Sharif Aly
Salomon Amar
Rama Amara
Gaya Amarasinghe
Khalid Amari
Richard Ambinder
Zandrea Ambrose
Amal Amer
Manuel Amieva
Rogiero Amino
Lynn Amon
Gregor Anderluh
Deborah Anderson
Gregory Anderson
David Andes
Felipe Andrade
Norma Andrews
Helene Andrews-Polymenis
Alex Andrianopoulos
Elliot Androphy
Matthew Angel
Mary Ann McDowell
Aftab Ansari
Rustom Antia
Lis Antonelli
Janis Antonovics
Takashi Aoki
Cristian Apetrei
Yiorgos Apidianakis
Victor Appay
James Aramini
Miguel Aranda
Jacques Archambault
José Argüello
Pablo Argüeso
Minetaro Arita
Yasuo Ariumi
Raffi Aroian
David Aronoff

Gustavo Arrizabalaga
James Arthos
Ann Arvin
Sassan Asgari
David Askew
David Asmuth
Aravind Asokan
Walter Atwood
Victoria Auerbuch
Yossef Av-Gay
Paul Axelsen
Janelle Ayres
Abdu Azad
Subash Babu
Steven Bachenheimer
Martin Bachmann
Steffen Backert
Vladimir Badovinac
Marc Baguelin
Justin Bahl
Yong-Sun Bahn
Joel Baines
Anna Bakardjiev
Andrew Baker
David Baker
Susan Baker
Tim Baker
Cosima Baldari
Jonathan Ball
Jimmy Ballard
David Baltrus
Charles Bangham
James Bangs
Ehud Banin
Lawrence Banks
James Bann
Shweta Bansal
Daniel L. Barber
Alan Barbour
A. Barclay
Wendy Barclay
Ralph Baric
Carolina Barillas-Mury
Eric Barklis
Dale Barnard
John Barnwell
Dan Barouch
Antonio Barragan
François-Xavier Barre
Ben Barres
Alan Barrett
Alyssa Barry
Clifton Barry
Peter Barry
Ralf Bartenschlager
Nathan Bartlett
Erik Barton
Christopher Basler
Paul Bates
Thomas Baumert
Andreas Baumler
David Baumler
Andreas Baur
Sina Bavari
Arnold Bayer
Peter Beard
Hans-Peter Beck
Niko Beerenwinkel
James Beeson
Samuel Behar
Marcel Behr
Sven-Erik Behrens
Eduardo Bejarano
George Belov
Alexia Belperron
Robert Belshaw
Eitan Ben-Dov
Chris Benedict
José Bengoechea
Lbachir BenMohamed
Richard Bennett
Barbara Bensing
Steven Bensinger
Leslie Berg
Rance Berg
Jeffrey Bergelson
Molly Bergman
Cornelia Bergmann
Carl Bergstrom
Andreas Bergthaler
Vincent Beringue
Ben Berkhout
Gerald Berkowitz
Judith Berman
Phillip Berman
Luiz Bermudez
Jürgen Bernhagen
Umberto Bertazzoni
Richard Bessen
Sonja Best
Michael Betts
Stephen Beverley

Sumita Bhaduri-McIntosh
Gyan Bhanot
Purnima Bhanot
Nina Bhardwaj
Tanmoy Bhattacharya
David Bhella
Vera Bianchi
Martine Biard-Piechaczyk
Paul Bieniasz
Oliver Billker
James Binley
Andrew Binns
Thomas Bjarnsholt
Pamela Bjorkman
Niklas Björkström
Samuel Black
Jenefer Blackwell
Ira Blader
Carol Blair
David Blair
Stéphane Blanc
Stephane Blanc
Steven Blanke
Joel Blankson
James Bliska
Joseph Bliss
Gary Blissard
Ian Blomfield
Bruno Blondel
David Bloom
Jesse Bloom
Adriano Boasso
Michael Boeckh
David Boehr
Teun Boekhout
Debby Bogaert
Matthew Bogyo
David Bolam
Blaise Boles
Michael Bolker
Melvin Bolton
David Boltz
Jennifer Bomberger
Morgane Bomsel
Ivo Boneca
Marcelo Bonini
Eliette Bonnefoy
Marc Bonten
André Boonstra
John Boothroyd
Mike Boots
Suresh Boppana
Seth Bordenstein
Kathleen Boris-Lawrie
Katherine Borkovich
Bradley Borlee
Georg Bornkamm
Eli Borrego
Eric Bortz
Irene Bosch
Jurgen Bosch
Santanu Bose
Helena Boshoff
Steven Bosinger
Katharine Bossart
Ken Bost
Fadila Bouamr
Michael Bouchard
Martin Boulanger
Steeve Boulant
Aida Bouratbine
Robert Bourret
Chris Boutell
Nicole Bouvier
Klaas Bouwmeester
Andrew Bowie
Scott Boyd
Jon Boyle
Thomas Braciale
Steven Bradfute
Kenneth Bradley
Peter Bradley
Jonathan Bramson
Curtis Brandt
Philip Branton
Jonathan Braun
Alejandra Bravo
Klaus Brehm
Jason Brenchley
Wade Bresnahan
Stephane Bressanelli
Luis Brieba
John Briggs
Volker Briken
David Briles
Paul Brindley
Frederic Bringaud
Melanie Brinkmann
Margo Brinton
Dustin Brisson
Anne Britt
William Britt

Robert Britton
Warwick Britton
Mark Brockman
Peter Brodersen
Priscille Brodin
Igor Brodsky
Barbara Broeker
Heike Broetz-Oesterhelt
Barbara Bröker
Frank Brombacher
David Brooks
Laurent Brossay
Alistair Brown
Charles Brown
Eric Brown
Gordon Brown
Judith Brown
Edward Browne
Petr Broz
John Brumell
Zabrina Brumme
Wolfram Brune
James Bryers
Mark Brynildsen
Peter Brzovic
Juliane Bubeck-Wardenburg
Susan Buchanan
Michael Buchmeier
Carmen Buchrieser
Christopher Buck
Frederick Buckner
Pierre Buffet
Marcus Buggert
Alexander Bukreyev
James Bull
Peter Bull
Andre Buret
Stacey Burgess
Jozsef Burgyan
Barbara Burleigh
James Burns
Dennis Burton
Benjamin Burwitz
Frederic Bushman
Jacinta Bustamante
Sarah Butcher
Geraldine Butler
Noah Butler
Gerald Byrne
Andrew Byrnes
Caughey Byron
Daniel C. Nelson
Richard Calderone
Richard Calendar
Guy Caljon
Niels Olsen Saraiva Câmara
Christian Cambillau
Caroline Cameron
Jennifer Cameron
Paul Cameron
Andrew Camilli
Gabriella Campadelli-Fiume
Daniel Campbell
Edward Campbell
Kerry Campbell
Thomas Campbell
Kenneth Campellone
Nathalie Campo
David Canaday
Bruno Canard
Paula Cannon
Edouard Cantin
Michael Caparon
Patrizia Caposio
Rey Carabeo
John Card
Rhonda Cardin
Cathleen Carlin
Jonathan Carlson
Fredric Carlsson
James Carlyle
Jason Carlyon
Elisabeth Carniel
Daniel Carr
John Carr
Mark Carrington
Nicola Carter
Jeffrey Cary
John Casey
James Cassat
Bastien Castagner
Araceli Castillo
José Caston
António Castro
Maria Castro
Robert Cattaneo
Simon Cauchemez
Francesc Cebrià
Thomas Cecere
Lynette Cegelski
Jean Celli
Stella Cesari

George Chaconas
Kris Chadee
Ann Chahroudi
Donald Champagne
Eric Chan
John Chan
Bala Chandran
Kartik Chandran
Jeff Chang
Kyong-Mi Chang
Theresa Chang
Moses Chao
Pratip Chattopadhyay
sujata Chaudhari
Linda Chelico
Benjamin Chen
Honglin Chen
Mei-Ru Chen
Sharon Chen
Zhixiang Chen
Hua Cheng
Bobby Cherayil
Peter Cherepanov
Yury Chernoff
Sara Cherry
Bruce Chesebro
Christophe Chevillard
John Chiorini
Chetan Chitnis
Michael Cho
Hyeryun Choe
Il-Ryong Choi
Nicolas Chomont
claire Chougnet
Gerardo Chowell
Dinesh Christendat
Neil Christensen
David Christianson
Peter Christie
Hong Wei Chu
June Kwon Chung
Luka Cicin-Sain
Andrea Cimarelli
Vincenzo Ciminale
Alexander Ciota
Paul Clapham
François Clavel
Steven Clegg
Jan Clement
Axel Cloeckaert
Joachim Clos
Christine Clouser
Gitta Coaker
Sarah Cobey
Ian Cockburn
Donald Coen
Tom Coenye
Jorn Coers
Gary Cohen
David Colby
Jonathan Coles
Jerome Collemare
Andrew Collins
James Collins III
Marco Colonna
Richard Compans
Teresa Compton
Wayne Conlan
Sarah Connolly
Mark Connors
Nicholas Conrad
Paul Converse
Klaus Conzelmann
Gregory Cook
Brad Cookson
Andrea Cooper
Chester Cooper
Christopher Cooper
Isabelle Coppens
Gordon Corey
Lawrence Corey
Brendan Cormack
Stephania Cormier
Cynthia Cornelissen
Nicolas Corradi
Laurent Coscoy
Pascale Cossart
Agnès Coste
Susan Cotmore
Peggy Cotter
Fasseli Coulibaly
Sarah Coulthurst
Kevin Couper
Antonello Covacci
Leah Cowen
Siobhan Cowley
Andrea Cox
Jeffery Cox
Brendan Crabb
Alister Craig
Lisa Craig
Robert Cramer

Alison Crawford
Alison Criss
Ileana Cristea
Paul Crocker
Peter Crompton
James Cronin
James Crowe
Angela Cruz
Laszlo Csonka
Jie Cui
Bryan Cullen
Paul Cullen
James Culver
Anthony Cunningham
Colm Cunningham
James Cunningham
Aubrey Cunnington
Christina Cuomo
Stephen Curry
Roy Curtiss III
Melanie Cushion
Jason Cyster
Clarissa da Costa
Akram Da'dara
Johanna Daily
Lisa Daley-Bauer
Tamas Dalmay
Marc Dalod
John Dalton
Satya Dandekar
Kenyon Daniel
Robert Daniels
Pranav Danthi
Richard D'Aquila
Richard Darveau
Gobardhan Das
Saumitra Das
Sandip Datta
Robert Daum
John David
L. David Sibley
David Davido
Stephen Davies
Brigid Davis
Mark Davis
Christopher Dawson
Thomas Dawson, Jr.
Brad Day
Cheryl Day
Patrick De Baetselier
Luiz Pedro de Carvalho
Raffele De Francesco
Cornelis de Haan
Sybren de Hoog
Juan C. de la Torre
Marianne De Paepe
Jaap de Roode
Aravinda de Silva
Petra de Verdier
Emmie de Wit
Marcel de Zoete
Ralph Dean
Zeger Debyser
Steven Deeks
George Deepe, Jr.
Victor DeFilippis
Raffaele DeFrancesco
Mariapia Degli-Esposti
Christoph Dehio
Kirk Deitsch
Hernando del Portillo
Henri-Jacques Delecluse
Neal Deluca
Eric Delwart
Christophe d'Enfert
Hongyu Deng
Mark Denison
Eric Denkers
Joe Dent
Dee Denver
Lisa Denzin
Rajendar Deora
Cynthia Derdeyn
Vojo Deretic
Isabelle Derré
Jeremy Derrick
Petra Dersch
Prashant Desai
Albert Descoteaux
David Deshazer
Alain Dessein
Andrea Dessen
Darrell Desveaux
Corrella Detweiler
Murray Deutscher
Rebekah DeVinney
Eric Déziel
Antonio Di Pietro
Francesco Di Serio
Gill Diamond
Arturo Diaz
Felipe Diaz-Griffero

Marcel Dicke
Martin Dickman
Andrew Diener
Binh Diep
Lars Dietrich
Stephanie Diezmann
Paul Digard
Joseph Dillard
Daniel DiMaio
Dimiter Dimitrov
Nigel Dimmock
George Dimopoulos
Charles Dinarello
Jianbing Ding
Rhoel Dinglasan
Francisco Dionisio
Dirk Dittmer
Ulf Dittmer
Maziar Divangahi
Narendra Dixit
Julianne Djordjevic
Andrzej Dlugosz
Thomas Dobner
Karen Dobos
Roberto Docampo
David Dockrell
Peter Dodds
Mike Doenhoff
Christian Doerig
T. Mark Doherty
Valerian Dolja
Lars Dölken
Robert Doms
Arjen Dondorp
Xinnian Dong
Ruben Donis
Denise Doolan
John Doorbar
Katie Doores
Kelly Doran
Philip Dormitzer
George DosReis
Angela Douglas
Simon Dove
Shaynoor Dramsi
Simon Draper
Michael Drebot
Christian Drosten
Peter Dube
David Dubnau
Jean Dubuisson
Jaquelin Dudley
Penelope Duerksen-Hughes
Siobain Duffy
Alain Dufour
Guillaume Dumenil
Eric Dumonteil
Joseph Duncan
Paul Dunman
Gary Dunny
Sarah Dunstan
Olivier Duron
Michael Dustin
Rebecca Dutch
Daniel Ebbole
Gregory Ebel
Hideki Ebihara
Joshua Eby
W. Edward Swords
Jennifer Edwards
Stacey Efstathiou
Laurence Eisenlohr
Amie Eisfeld
Nels Elde
John Elder
Ioannis Eleftherianos
Santiago Elena
Ayman El-Guindy
Richard Elliott
Jeff Ellis
Jerrold Ellner
Michael Emerman
Stephane Emiliani
Alan Engelman
David Engman
Christian Engwerda
Luis Enjuanes
Jost Enninga
Lynn Enquist
Alexander Ensminger
Armin Ensser
Mark Eppinger
Joel Ernst
Robert Ernst
Peter Espenshade
Eduardo Espeso
Jerome Estaquier
José Esté
Mariano Esteban
Jacob Estes
David Evans
Jay Evans

Matthew Evans
David Everly
Mario Fabri
Anna-Bella Failloux
Erik Falck-Pedersen
Franco Falcone
Bryce Falk
Padraic Fallon
Ferric Fang
Donna Farber
Stephen Farrand
Helen Farrell
Michael Farzan
Bruno Favery
Michael Federle
Mario Feldman
Heinz Feldmann
Carl Feng
Ningguo Feng
Pinghui Feng
Ana Fernandez-Sesma
Guido Ferrari
Martin Ferris
Tobias Feuchtinger
Paul Fey
Richard Ffrench-Constant
Mark Field
Joshua Fierer
Luisa Figueiredo
Sergio Filipe
Scott Filler
Alain Filloux
Claire Marie Filone
Keisha Findley
Stefan Finke
Matthew (Mat) Fisher
Katherine Fitzgerald
Louis Flamand
Bernhard Fleckenstein
James Fleckenstein
Suzanne Fleiszig
Erik Flemington
Michelle Flenniken
S. Flint
Alexander Flügel
Ervin Fodor
Ismael Fofana
Antonella Folgori
J. Forrest
Naomi Forrester
Elizabeth Fortunato
Sarah Fortune
Simon Foster
Ron Fouchier
Keith Fowke
Vance Fowler
Cornel Fraefel
Nicole Frahm
Nathalie Franc
Olivera Francetic
Dara Frank
Gad Frankel
Michael Franklin
James Fraser
John Fraser
Martin Fraunholz
Eric Freed
Nancy Freitag
Daved Fremont
Martyn French
Ute Frevert
Harvey Friedman
Matthew Frieman
Paul Friesen
Timothy Friesen
Teresa Frisan
Friedrich Frischknecht
Rémi Fronzes
Horacio Frydman
Tobias Fuchs
Robert Fujinami
Takashi Fujita
Dahlene Fusco
Ruth Gabizon
Michaela Gack
Jennifer Gaddy
Pascal Gagneux
Michael Gale, Jr.
Thomas Gallagher
Teresa Gallart
Giorgio Gallinella
Richard Gallo
Jean-Luc Gallois
Denise Galloway
Shou-Jiang Gao
Zhiyong Gao
Laurent Gapin
David Garboczi
Robert Garcea
Felipe Garcia
J. Victor Garcia
Juan Antonio García

Mariano Garcia-Blanco
Adolfo Garcia-Sastre
Dominique Garcin
Melanie Gareau
Suzanne Garland
Danielle Garsin
Nicholas Gascoigne
Walter Gassmann
Pablo Gastaminza
William Gause
Gregory Gauthier
Timothy Geary
Adam Geballe
Suzanne Geerlings
Teunis Geijtenbeek
Thomas Geisbert
Caroline Genco
Howard Gendelman
Elke Genersch
George Georgiou
Ronald Germain
Paul Gershon
Antoine Gessain
Elodie Ghedin
Frank Gherardini
Godelieve Gheysen
Partho Ghosh
Mauro Giacca
Chou-Zen Giam
Luis Giavedoni
David Giedroc
Robert Gifford
Tim Gilberger
Jason Gill
Sarjeet Gill
Sarah Gilmore
Michael Ginger
Ole Gjoerup
Britt Glaunsinger
Michael Glickman
Michael Glogauer
Jerome Goddard
Paul Goepfert
Stephen Goff
Marielle Gold
Scott Gold
Joanna Goldberg
Marcia Goldberg
William Goldman
Pascal Goldschmidt-Clermont
Tatyana Golovkina
David Gondek
Fernando Gonzalez-Candelas
Mercedes Gonzalez-Juarrero
Felicia Goodrum
Paul Gorry
Susan Gottesman
Geoffrey Gottlieb
Heinrich Gottlinger
Eva Gottwein
Friedrich Götz
David Grainger
Arash Grakoui
Lone Gram
Thomas Gramberg
Roger Grand
Duane Grandgenett
Neil Granger
Sarah Grant
Warwick Grant
Clive Gray
Urs Greber
Jeffrey Green
Patrick Green
Sharone Green
William Green
Doron Greenbaum
Peter Gresshoff
Christoph Grevelding
Giorgio Gribaudo
Anne Griep
Diane Griffin
Paul Griffiths
Michael Grigg
Sergio Grinstein
Tamara Gritsun
Eduardo Groisman
Allison Groseth
Jeff Grotzke
Liber Grubhoffer
Angelika Grundling
Christoph Grundner
Kay Grunewald
Marc-Jan Gubbels
Christophe Guilhot
David Guiliano
Karen Guillemin
Nancy Guillen
Keith Gull
Suryaram Gummuluru
John Gunn
Arthur Gunzl

Haitao Guo
Ju-Tao Guo
Sunetra Gupta
Ricardo Gürtler
Esteban Gurzov
Kurt Gustin
Bart Haagmans
David Haake
Hubertus Haas
Rainer Haas
Ashley Haase
Georg Häcker
Ted Hackstadt
Elias Haddad
Julius Clemence Hafalla
Ilka Haferkamp
Young Hahn
Matthais Hahn
David Haig
Nancy Haigwood
Stephen Hajduk
Rana Hajjeh
Mohamed Hakimi
Morgan Hakki
Benjamin Hale
William Halford
Andrew Hall
Jessica Hamerman
Sven Hammerschmidt
Wolfgang Hammerschmidt
Jiahuai Han
Lynn Hancock
Dorit Hanein
Diana Hansen
Immo Hansen
Alexandre Harari
Kim Hardie
Clifford Harding
Wolf-Dietrich Hardt
Richard Hardy
Edward Harhaj
David Harris
Mark Harris
NIcola Harris
Reuben Harris
Joe Harrison
Paul Harrison
Robert L. Harrison
Stephen Harrison
Axel Hartke
Dominik Hartl
Elizabeth Hartland
Susanne Hartmann
Kevan Hartshorn
John Harty
Ronald Harty
Eric Harvill
Caroline Harwood
Rie Hasebe
Masanori Hatakeyama
Alan Hauser
Susanne Häussler
Thomas Hawn
Barton Haynes
S. Hayward
Bin He
Chuan He
Cynthia He
Ping He
Mark Head
Kevin Healy
Patrick Hearing
William Heath
Nicholas Heaton
Jürgen Heesemann
Johannes Hegemann
Bernd Heimrich
Manfred Heinlein
David Heinrichs
Franz Heinz
Robert Heinzen
Mark Heise
Joseph Heitman
Zdenek Hel
Ekaterina Heldwein
Ari Helenius
John Helmann
Helena Helmby
Piers Hemsley
Els Henckaerts
Robert Hendricks
Hartmut Hengel
Philipp Henneke
Thomas Henry
Scott Hensley
Joshua Herbeck
Debroski Herbert
Elisabeth Herniou
Susanne Herold
Anat Herskovits
Laura Hertel
Heiko Herwald

Dagmar Heuer
Holger Heuer
Tarek Hewezi
James Hewitson
Merilyn Hibma
Andres Hidalgo
Hubert Hilbi
William Hildebrand
Rolf Hilgenfeld
Ann Hill
Darryl Hill
Julian Hillyer
Catarina Hioe
Alec Hirsch
Vanessa Hirsch
Robert Hirt
John Hiscott
Amy Hise
Andrew Hislop
Florian Hladik
James Hoch
Richard Hodinka
Johan Hofkens
Daniel Hoft
Cory Hogaboam
Deborah Hogan
Saskia Hogenhout
Brenda Hogue
Tobias Hohl
Thomas Hohn
Michael Holbrook
David Holden
Lindy Holden-Dye
Anthony Holder
Edward Hollox
Edward Holmes
Fred Homa
Dirk Homann
Derek Hood
Magnus Hook
Kelli Hoover
Thomas Hope
Lien-I Hor
Masayuki Horie
Yasuhiko Horiguchi
David Horn
Stacy Horner
Mady Hornig
William Horsnell
Alexander Horswill
Branka Horvat
Curt Horvath
Anne Hosmalin
Eileen Hotze
Fanjian Hou
Barbara Howlett
Peter Howley
Marc Hoylaerts
Peter Hraber
Michael Hsieh
Bin Hu
Ke Hu
Wei-Shau Hu
I-Chueh Huang
Jinghe Huang
Xi Huang
Andree Hubber
Bernhard Hube
Alisa Huffaker
Gary Huffnagle
David Hughes
Kelly Hughes
Stephen Hughes
Raymond Hui
Edgar Huitema
Christina Hull
Scott Hultgren
Chiung-Yu Hung
Deborah Hung
David Hunstad
Peter Hunt
Christopher Hunter
Eric Hunter
Martha Hunter
Nora Hunter
William Hunter
Jason Huntley
Laurence Hurley
Greg Hurst
Aeron Hurt
Eric Huseby
Lindsey Hutt-Fletcher
Alexander Idnurm
Tetsuro Ikegami
Jean-Luc Imler
Hanne Ingmer
Naohiro Inohara
Naoki Inoue
David Irani
Javier Irazoqui
Keith Ireton
Takashi Irie

James Ironside
Stuart Isaacs
Ralph Isberg
Ken Ishii
Masayuki Ishikawa
Stanimir Ivanov
Juraj Ivanyi
Tina Iverson
Yoshihiro Izumiya
Andrew Jackson
William Jackson
Jeffrey Jacobson
John Jaenike
Chinnaswamy Jagannath
Sanjay Jain
Sirpa Jalkanen
Leo James
Timothy James
Julie Jameson
Marc Jamin
Guilhem Janbon
McKeating Jane
Dragana Jankovic
Barbara Jarausch
Theodore Jardetzky
Dan Jarosz
Ronald Javier
F. Javier Cabañes
Max Jean Toledo
Lars Jelsbak
Urs Jenal
Stephen Jenkins
Howard Jenkinson
Annette Jensen
Mark Jepson
Ann Jerse
Samithamby Jeyaseelan
Ravi Jhaveri
Yinduo Ji
Lubin Jiang
Zhengfan Jiang
Francis Jiggins
Xia Jin
Ludger Johannes
Eric Johannsen
Michael Johansson
Chandy John
Alexander Johnson
David Johnson
Karyn Johnson
Marc Johnson
R Johnson
R. Paul Johnson
Welkin Johnson
Brad Jones
Clinton Jones
Ian Jones
Jeff Jones
Malcolm Jones
Michael Jones
Stipan Jonjic
Matthieu Joosten
Catherine Jopling
Christine Josenhans
Sarah Joseph
Jean-Philippe Julien
Isabelle Jupin
Dominika Jurkovic
Pradeep Kachroo
Aras Kadioglu
Susan Kaech
Jonathan Kagan
Kevin Kain
Robert Kalejta
Isgouhi Kaloshian
Nobuhiko Kamada
Seogchan Kang
Michael Kann
Thirumala-Devi Kanneganti
Michael Kanost
Ray Kaplan
Stefan Kappe
William Karesh
David Karlin
Annika Karlsson
Gunilla Karlsson Hedestam
Jonathan Karn
Timothy Karr
Denuja Karunakaran
Fatah Kashanchi
Richard Kaslow
Fumiaki Katagiri
Christine Katlama
Peter Katsikis
Richard Katz
Michael Katze
Daniel Kaufmann
Stefan Kaufmann
Rupert Kaul
Amitinder Kaur
Deepak Kaushal
Radhey Kaushik

Shun-ichiro Kawabata
Ikuro Kawagishi
Toshiaki Kawakami
Thomas Kawula
Michael Kay
Kenneth Kaye
Paul Kaye
Joseph Keane
Katherine Kedzierska
Brandon Keele
Scott Keely
Marijke Keestra
Thomas Kehl-Fie
Alison Kell
Paul Kellam
Nancy Keller
Michelle Kelliher
John Kelly
Brian Kelsall
Christopher Kemball
Eric Kemen
Melissa Kendall
Linda Kenney
Stephen Kent
Thomas Kepler
Robin Ketteler
Vineet KewalRamani
Nemat Keyhani
Imtiaz Khan
Kamal Khanna
Rajiv Khanna
Hossein Khiabanian
Alexander Khromykh
Elliott Kieff
Margaret Kielian
Marjolein Kikkert
Byung Kim
Carol Kim
Charles Kim
Dennis Kim
Peter Kima
Paul Kinchington
Jason Kindrachuk
Christine King
Christopher* King
Irah King
Kayla King
Nicholas King
Frank Kirchhoff
Karla Kirkegaard
Hirohito Kita
Scott Kitchen
Todd Kitten
Tohru Kiyono
Per Johan Klasse
Nichole Klatt
Colin Kleanthous
Bruce Klein
Robyn Klein
Aloysius Klingelhutz
Kimberly Klonowski
Rob Knell
David Knipe
Leigh Knodler
Dennis Ko
Joachim Koch
Georg Kochs
Uwe Koedel
Theresa Koehler
David Koelle
Leo Koenderman
Renate Koenig
Alain Kohl
Nikolay Kolev
Jay Kolls
Dennis Kolson
Kouacou Konan
Leopold Kong
Michael Konkel
James Konopka
Michael Koomey
Eugene Koonin
Geert Kops
Bette Korber
Hardy Kornfeld
Benoit Kornmann
Nicole Koropatkin
Konstantin Korotkov
Anita Koshy
Sergei Kotenko
Andrew Kotze
Richard Koup
Konstantin Kousoulas
Igor Kovalchuk
Timothy Kowalik
Yoshio Koyanagi
Joanna Koziel
Pamela Kozlowski
Peter Kraiczy
Florian Krammer
Sven Krappmann
Laurent Kremer

Thomas Krey
Lakshmi Krishnan
Bernhard Krismer
Thomas Kristie
Mitchell Kronenberg
James Kronstad
Pascale Kropf
Anne Krug
Laurie Krug
Damian Krysan
Urszula Krzych
Paul Kubes
Tomoko Kubori
Karl Kuchler
Meta Kuehn
Ralf Kueppers
Richard Kuhn
Carol Kumamoto
Pankaj Kumar
Purnima Kumar
Barbara Kunkel
Jonathan Kurtis
Christian Kurts
Sergei Kusmartsev
Olaf Kutsch
Kazuhiro Kutsukake
Mamuka Kvaratskhelia
Douglas Kwon
Peter Kwong
Fabien Labroussaa
Andrew Lackner
Benoit Lacroix
D. Borden Lacy
Monique Lafon
Bernard Lafont
Michael Lagunoff
Timothy Lahey
Erh-Min Lai
Michael M. C. Lai
Lou Laimins
Jean-François Laliberté
Joseph Lam
Tracey Lamb
Kris Lambert
Paul Lambert
Louis Lambrechts
David Lambright
Ke Lan
Christina Lancioni
Nathaniel Landau
Thomas Lane
Jeroen D Langereis
Jan Langeveld
Gordon Langsley
Lewis Lanier
Iñigo Lasa
Corinne Lasmezas
Jean-Paul Latgé
Wyndham Lathem
Georg Lauer
Adam Lauring
Grégoire Lauvau
Catherine Lavazec
Mara Lawniczak
Brian Lazzaro
Jacques Le Pendu
Susan Lea
Brian Leander
Francois Lebreton
Michael Lederman
Benhur Lee
Bok-Luel Lee
Chia Lee
Jean Lee
Peter Lee
Vincent Lee
Yong-Hwan Lee
Molly Leecaster
Ann Leen
Kevin Legge
Nicolas Legrand
Paul Lehner
David Leib
Salome Leibundgut-Landmann
Andrew Leigh Brown
Philippe Lemey
Stanley Lemon
Deborah Lenschow
Laurel Lenz
John Leong
Philip Leopold
Jean Lepault
Didier Lereclus
Ganjana Lertmemongkolchai
Julien Lescar
Justin Lessler
Elena Levashina
Stuart Levitz
Virgilio Lew
Shawn Lewenza
Sharon Lewin
David Lewinsohn

George Lewis
Jianrong Li
Mei-Ling Li
Ming Li
Xiao-Dong Li
Xin Li
Yi Li
Chen Liang
T. Jake Liang
Mathias Lichterfeld
Brian Lichty
Paul M Lieberman
Susan Liebman
Thomas Liebrand
Egil Lien
Jan Liese
Mark Liles
Maria Limberis
Na-Sheng Lin
Rongtuan Lin
Wenyu Lin
Xiaorong Lin
Xin Lin
Zhen Lin
Paul Ling
Klaus Lingelbach
Dirk Linke
Michail Lionakis
Volker Lipka
Su Lishan
Susan Little
Fenyong Liu
George Liu
Haoping Liu
Jie Liu
Shan-Lu Liu
Yancheng Liu
Yule Liu
Zheng-Gang Liu
Manuel Llinás
Kwok-Wai Lo
Mario Lobigs
Volker Lohmann
James Lok
P'ng Loke
Steven Lommel
Ben Longdon
Yueh-Ming Loo
Susana Lopez
Carolina López
Miguel López-Botet
Juan Jose Lopez-Moya
Jose Lopez-Ribot
Graciela Lorca
Michael Lorenz
Jennifer Loros
Alex Loukas
Sebastian Lourido
Philip LoVerde
Anice Lowen
Pedro Lowenstein
Laura Lowery
Franklin Lowy
Olga Lubman
Stephen Luby
Stephan Ludwig
Micah Luftig
David Lukac
Aron Lukacher
Nicholas Lukacs
Julius Lukes
Oana Lungu
Guangxiang Luo
Sara Lustigman
Katherine Luzuriaga
Samantha Lycett
Joseph Lynch
Jiyan Ma
Wenbo Ma
Zhonghua Ma
Margaret MacDonald
Michael Mach
Fabiana Machado
Carolyn Machamer
Matthias Machner
Jason Mackenzie
David Mackey
Erich Mackow
James MacLachlan
Annette MacLeod
Katherine MacNamara
Hiten Madhani
Carsten Magnus
Thien-Fah Mah
Renaud Mahieux
Martin Maiden
Rick Maizels
Kristiina Makinen
Shinji Makino
Natalia Malachowa
Emilio Malchiodi
Frank Maldarelli

Ray Malfavon-Borja
Harmit Malik
J.S. Malik Peiris
Richard Malley
Carolyn Malmstrom
Julin Maloof
Francois Malouin
Ofer Mandelboim
Micahel Mandell
Nicolas Manel
Nicholas Maness
Balaji Manicassamy
Shannon Manning
John Mansfield
Jean Manson
Daniel Mansur
Pierre-Yves Mantel
Yuxin Mao
Giulia Marchetti
Antonio Marcilla
Joseph Marcotrigiano
David Margolis
David Margulies
G. Maria Hansch
Brian Mark
R. Mark Buller
Melanie Marketon
Thomas Marlovits
Ernesto Marques
Carl Marrs
Mark Marsh
Eric Martens
Matthias Marti
Coralie Martin
Gregory Martin
Malcolm Martin
Richard Martin
Stephen Martin
Elena Martinelli
Juan Martinez
Encarnacion Martinez-Salas
Luis Martínez-Sobrido
Juan Martin-Serrano
John Mascola
Pietro Mastroeni
Maria Masucci
Candace Mathiason
Cyrille Mathieu
Frederick Matsen
Masao Matsuoka
Joseph Mattapallil
Bonazzi Matteo
Joerg Mattes
Keith Matthews
Qiana Matthews
Stephen Matthews
Seema Mattoo
Kai Matuschewski
Wendy Maury
Josef Mautner
Robin May
Andreas Mayer
Katrin Mayer-Barber
Didier Mazel
Joseph McArdle
Alison McBride
Jere McBride
Dennis McCance
Douglas McCarty
James McCaw
Mark McClain
Bruce McClane
Malcolm McConville
Craig McCormick
Jonathan McCullers
Joseph McCune
Kathleen McDonough
John McDowell
Johnjoe McFadden
Dorian McGavern
Michael McHeyzer-Williams
Gerald McInerney
Kevin McIver
Jane McKeating
Lyle McKinnon
Margaret McKinnon
James McLachlan
John McLauchlan
Jason McLellan
Rima McLeod
Rachel McLoughlin
Aoife McLysaght
Don McManus
Andrew McMichael
Rachel McMullan
Eileen McNeill
Henry McSorley
Stephen McSorley
Michael McVoy
Joan Mecsas
Jean-Louis Mege
Borna Mehrad

Annemarie Meijer
Graeme Meintjes
Marina Meixner
John Mekalanos
Peter Melby
Thomas Melendy
Jose Melero
Gregory Melikyan
J. L. Mellies
Cicero Mello
Cécile Ménez
Gustavo Menezes
Tesfaye Mengiste
Joris Menten
Jason Mercer
Enrique Mesri
Martin Messerle
Dennis Metzger
Edward Miao
Jun Miao
Michelle Michalski
Kristin Michel
Jan Michiels
Tam Mignot
Ivan Mijakovic
W. Allen Miller
Cathy Miller
Christopher Miller
George Miller
Samuel Miller
Virginia Miller
William Miller
Kathryn Miller-Jensen
Kingston Mills
Booki Min
Baruch Minke
Olivo Miotto
Aaron Mitchell
Patrick Mitchell
Thomas Mitchell
Tim Mitchell
Makedonka Mitreva
Masaaki Miyazawa
Valerie Mizrahi
Harry Mobley
Edward Mocarski
Yorgo Modis
Jeremy Modridge
Christopher Mody
Axel Mogk
Ian Mohr
Susan Moir
Denise Monack
Skorn Mongkolsuk
Luis Montaner
Cesare Montecucco
David Montefiori
Ronald Montelaro
Cary Moody
Hannah Moore
Julie Moore
Martin Moore
Patrick Moore
Penny Moore
Nathaniel Moorman
Darius Moradpour
Miguel Morales
Thomas Moran
Kevin Morano
Slivia Moreno
Christopher Mores
Sarah Morgan
Koki Morizono
Renato Morona
Jack Morris
Richard Morris
Liam Morrison
Thomas Morrison
Joachim Morschhäuser
Stephen Morse
Nathan Mortimer
George Mosialos
Bernard Moss
David Mosser
Karen Mossman
Serge Mostowy
Walther Mothes
Jeremy Mottram
Zhirong Mou
Andrew Mouland
Adrian Mountford
Hugo Mouquet
Benoit Moury
Laurence Mouton
Anne Mueller
David Mueller
Ivo Mueller
Scott Mueller
Christian Muenz
Martin Muggeridge
Shaeri Mukherjee
Rita Mukhopadhyay

Suchetana Mukhopadhyay
Viktor Muller
William Muller
Marcel Müller
Mathias Müller
Viktor Müller
Matt Mulvey
Matthew Mulvey
Ulrike Munderloh
Joshua Munger
Carol Munro
Christian Munz
Pablo Murcia
Thomas Murooka
Eain Murphy
Peter Murray
Susan Murray
Johannes Müthing
Nick Muzyczka
Mary Myerscough
Peter Myler
Joe Mymryk
Indira Mysorekar
Florian Nachon
Hiroki Nagai
Mojgan Naghavi
Shalin Naik
Meera Nair
Shigetou Namba
Franz Narberhaus
Keiko Naruse
Michael Nassal
Sheila Nathan
William Nauseef
Hans Nauwynck
William Navarre
Lionel Navarro
Miguel Navarro
Stuart Neil
Frank Neipel
Jay Nelson
Martha Nelson
Glen Nemerow
Mihai Netea
Karla Neugebauer
Gabriele Neumann
Peter Neumann
Christine Neuveut
Michael Nevels
Cedric NEVEU
Irene Newton
Olivier Neyrolles
Max Nibert
John Nicholas
Tracy Nicholson
Anthony Nicola
Annette Niehl
Christina Nielsen-Leroux
Stefan Niewiesk
Dimitar Nikolov
Falk Nimmerjahn
Shunbin Ning
Hiroshi Nishiura
Suzanne Noble
Takeshi Noda
Pauline Nol
Marina Noris
Karen Norris
Steven Norris
Joshua Nosanchuk
Mairi Noverr
Bogdan Nowicki
Tomoyoshi Nozaki
Jack Nunberg
Thorsten Nurnberger
Thomas Nutman
Patricia Nuttall
Joshua Obar
Marygorret Obonyo
Meagan O'Brien
Tom Obrig
David O'Connor
Roberta O'Connor
Eamon O'Dea
Una O'Doherty
Anne O'Garra
Kristen Ogden
Tomoaki Ogino
Amanda Oglesby-Sherrouse
Peter O'Hare
Päivi Ojala
Andrew Olive
Martin Olivier
Ken Olson
Michal Olszewski
Peng Kai Ong
Akira Ono
Peter Openshaw
Fred Opperdoes
Carlos Orihuela
Mary O'Riordan
Amos Orlofsky

David Ornelles
Kim Orth
Juan Ortín
Nir Osherov
Taku Oshima
Hiroyuki Oshiumi
Mike Osta
Nikolaus Osterrieder
Helena Ostolaza
Mario Ostrowski
Karen Ottemann
Michael Otto
Jing-hsiung James Ou
Marc Ouellette
Annette Oxenius
Michelle Ozbun
Oliver Pabst
Marie-Hélène Paclet
Slobodan Paessler
Antonio Pagán
Israel Pagán
Joseph Pagano
Antony Page
Rebecca Page
Emil Pai
Mirko Paiardini
Peter Palese
S Pallikkuth
Massimo Palmarini
Ann Palmenberg
Guy Palmer
Søren Paludan
Quintin Pan
Gianfranco Pancino
Pushpa Pandiyan
Yuan-Ping Pang
Santosh Panjikar
Ralph Panstruga
Giuseppe Pantaleo
Barbara Papadopoulou
Venizelos Papayannopoulos
James Papin
Vijayapalani Paramasivan
Gregory Pari
Dane Parker
Jane Parker
Michael Parker
John Parkinson
Robin Parks
Colin Parrish
Marilyn Parsons
Vinay Pathak
James Paton
John Patton
Rick Paul
W Paul Duprex
Silke Paust
Martin Pavelka
Christopher Peacock
Edward Pearce
Joao Pedra
Mario Pedraza-Reyes
R. Peebles, Jr.
Mark Peeples
Olve Peersen
Andrew Pekosz
You-Liang Peng
Carlos Penha-Goncalves
Francois Penin
Marion Pepper
Markus Perbandt
Miercio Pereira
Rushika Perera
Christian Perez
John Perfect
Stanley Perlman
Robert Perry
Katarina Persson
Andreas Peschel
Everett Pesci
Brian Peters
Morten Petersen
Ellena Peterson
Constantinos Petrovas
Julie Pfeiffer
Jennifer Philips
Lucia Piacenza
Andreas Pichlmair
David Pickup
Laura Piddock
Pedro Piedra
Gerald Pier
Susan Pierce
Philippe Pierre
Ted Pierson
Joseph Piesman
Thomas Pietschmann
Vincent Piguet
Satish Pillai
Zachary Pincus
Richard Pine
Jaume Pinol

David Pintel
James Pipas
Bodo Plachter
Vicente Planelles
Gregory Plano
Richard Plemper
Hidde Ploegh
Alexander Ploss
Christopher Plowe
Eric Poeschla
Stefan Pohlmann
Guido Poli
Sergei Kosakovsky Pond
Miquel Pons
Mikhail Pooggin
Leo Poon
Michel Popoff
Steven Porcelli
Daniel Portnoy
Jan Potempa
Wayne Potts
Michael Povelones
Gianni Pozzi
Gabriele Pradel
Reinhild Prange
Bidadi Prasad
Peter Prevelige
Lance Price
Alice Prince
Immo Prinz
Suzette Priola
Richard Proctor
David Proud
Patrick Provost
Jude Przyborski
Anne Puel
Jose Puente
Nathalie Pujol
Bali Pulendran
Mirja Puolakkainen
Georgiana Purdy
Lei Qi
Jin-Long Qiu
Feng Qu
Lee Quinton
Nancy Raab-Traub
Vincent Racaniello
Justin Radolf
Magdalena Radwanska
Manuela Raffatellu
Malini Raghavan
Laurence Rahme
Glenn Rall
Katherine Ralston
Paul Ramsland
Glenn Randall
Richard Randall
Troy Randall
Felix Randow
A.L.N. Rao
Rama Rao Amara
Didier Raoult
Chad Rappleye
Jason Rasgon
David Rasko
Poonam Rath
Phil Rather
Adam Ratner
Lee Ratner
Stefan Raunser
William Rawlinson
Ranjit Ray
Ratna Ray
Stuart Ray
Julian Rayner
Laurie K Read
Sullivan Read
Patrick Reading
Walt Ream
Mathias Reddehase
Alec Redwood
Sarah Reece
Douglas Reed
Michael Reed
Sharon Reed
Michael Reese
R. Keith Reeves
Fulvio Reggiori
Roland Regoes
Barbara Rehermann
Nancy Reich
Jonathan Reichner
Alan Rein
Neil Reiner
Celso Reis
Russel Reiter
David Rekosh
David Relman
Edmond Remarque
Han Remaut
Jyothi Rengarajan
Laurent Rénia

Rolf Renne
Natalia Requena
Félix Rey
Hugh Reyburn
Matthew Reynolds
Stuart Reynolds
Todd Reynolds
Kyu Rhee
Carlo Riccardi
Andrew Rice
Stephen Rice
Anthony Richardson
Douglas Richman
Sara Richter
Alan Rickinson
Michelle Riehle
Angelika Riemer
Jan Riemer
Arne Rietsch
Eleanor Riley
James Riley
Steven Riley
Guus Rimmelzwaan
Meritxell Riquelme
Jean Ristaino
Beatrice Riteau
Christophe Ritzenthaler
Susana Rivas
Amariliz Rivera
Frazer Rixon
Silke Robatzek
Carlos Robello
David Roberts
Richard Roberts
Erle Robertson
Rosemary Rochford
Daniel Rockey
Barry Rockx
Richard Roden
Isabel Roditi
Nuri Rodriguez
Maria Rodríguez
John Roehrig
David Rogers
Stephen Rogerson
John Rohde
George Rohrmann
Antonis Rokas
Richard Roller
Jeffrey Rollins
Chiara Romagnani
Ann Roman
Ute Römling
Keshet Ronen
Lijun Rong
Roy Roop II
Marilyn Roossinck
Carlos Ros
Patricia Rosa
Adriana Rosato
Jason Rosch
Susan Rosenberg
Ilan Rosenshine
Philip Rosenthal
Susan Ross
Michael Rossmann
Simon Rothenfusser
Carla Rothlin
Brice Rotureau
June Round
Barry Rouse
Thierry Rouxel
Aileen Rowan
Alex Rowe
Martin Rowe
Sarah Rowland-Jones
David Rowlands
Chad Roy
Craig Roy
Syamal Roy
Peter Rubenstein
Harvey Rubin
Daniel Rubio
Gloria Rudenko
Kendra Rumbaugh
Andreas Rummel
Jan Rupp
Steffen Rupp
Laura Rusche
Bruce Russell
Charles Russell
Colin Russell
David Russell
H. Ruth Ashbee
Julian Rutherford
Zsolt Ruzsics
Brent Ryckman
Bernhard Ryffel
Wang-Shick Ryu
Suraj Sable
Jonah Sacha
George Sachs

Ivan Sadowski
Jeroen Saeij
Xavier Saelens
Asier Sáez-Cirión
Saveez Saffarian
David Safronetz
Manish Sagar
Helen Saibil
Linda Saif
Takeshi Saito
Uma Sajjan
Juan Salazar
Shahram Salek-Ardakani
Marco Salemi
Padmini Salgame
Maxim Salganik
Nita Salzman
Clare Sample
Jeffery Sample
Charles Samuel
James Samuel
Rogier Sanders
Rozanne Sandri-Goldin
Hélène Sanfaçon
Rafael Sanjuan
Andrea Sant
Mario Santiago
Kaustuv Sanyal
Erica Saphire
Martin Sapp
Saumendra Sarkar
Peter Sarnow
Christopher Sassetti
Zsuzsanna Sasvari
Karla Satchell
Noah Sather
Sachiko Sato
Yorifumi Satou
Quentin Sattentau
John-Demian Sauer
Karin Sauer
Robert Sauerwein
Charles Saunders
Dorothea Sawicki
Charles Scanga
Vincenzo Scarlato
Joseph Schacherer
Brian Schaffhausen
Luis Schang
Julio Scharfstein
Hermann Schätzl
Mario Schelhaas
Mark Schembri
Cara-Lynne Schengrund
Sergio Schenkman
Dieter Schifferli
Adam Schikora
Stefan Schild
Martin Schlee
Mark Schleiss
Todd Schlenke
Larry Schlesinger
Patrick Schloss
Kimberly Schluns
Connie Schmaljohn
Sandra Schmid
Gudula Schmidt
M. Schmidt
Nathan Schmidt
Sarah Schmidt
Anthony Schmitt
Gerold Schmitt-Ulms
Mirco Schmolke
Michael Schmueck
Dirk Schnappinger
Achim Schnaufer
Anette Schneemann
Olaf Schneewind
Andre Schneider
David Schneider
Matthias Schnell
John Schoggins
Herman Scholthof
Christoph Schön
Jeffrey Schorey
Tony Schountz
Anthony Schryvers
Soeren Schubert
Christoph Schuller
Stephanie Schüller
Stacey Schultz-Cherry
Thomas Schulz
Erwin Schurr
Robert Schuurink
William Schwan
Ira Schwartz
Olivier Schwartz
Evelin Schwarzer
Martin Schwemmle
Kristin Scott
Phillip Scott
Rona Scott

Thomas Scott
D. Scott Merrell
Gavin Screaton
Eileen Scully
Matthew Seaman
Michael Seaman
Nabila Seddiki
Robert Seder
Jessica Seeliger
Brahm Segal
Gil Segal
Stephan Seiler
Ulrike Seitzer
Rafick-Pierre Sekaly
Liisa Selin
Barbara Selisko
Timothy Sellati
Bert Semler
Oliver Semmes
Adrish Sen
Ganes Sen
Laura Serbus
Irini Sereti
Lena Serghides
Janakiram Seshu
Peter Setlow
Alessandro Sette
Stephanie Seveau
David Severson
Karl Seydel
William Shafer
Yechiel Shai
Libo Shan
Feng Shao
Michael Shapira
Michal Shapira
Amit Sharma
Cynthia Sharma
Pushkar Sharma
Neelam Sharma-Walia
Lindsey Shaw
Megan Shaw
Pang-Chui Shaw
Dmitry Shayakhmetov
Lilach Sheiner
Aimee Shen
Li Shen
Qian-Hua Shen
Don Sheppard
Alan Sher
Philip Sherman
Ethan Shevach
Meiqing Shi
Pei-Yong Shi
Y. Shi
Naoto Shibuya
Chiaho Shih
Shin-Ru Shih
Michael Shiloh
Hiroyuki Shimizu
Toshiyuki Shimizu
Haina Shin
Sunny Shin
Joanna Shisler
Pranav Shivakumar
Charles Shoemaker
Naglaa Shoukry
Joshua Shrout
Deepak Shukla
Carol Hopkins Sibley
Shahid Siddique
Aleem Siddiqui
Inga Siden-Kiamos
Luis Sigal
Christina Sigurdson
Anita Sil
Robert Siliciano
Neal Silverman
Olivier Silvie
Valerie Sim
Cauchemez Simon
Scott Simon
Viviana Simon
Carmen Simón-Mateo
Alison Sinclair
John Sinclair
Neena Singh
Photini Sinnis
Eric Skaar
Rebecca Skalsky
Robert Skov
Jacek Skowronski
Betty Slagle
Mariel Slater
Barton Slatko
Mark Slifka
Pamela Small
Mark Smeltzer
James Smiley
Jolanda Smit
Duncan Smith
Everett Smith

Geoffrey Smith
Janet Smith
Joe Smith
Judith Smith
Terry Smith
Thomas Smithgall
Eric Snijder
Georges Snounou
Christopher Snyder
Magdalene So
Miguel Che Soares
Milena Soares
Peter Soboslay
Beate Sodeik
Kenneth Söderhäll
Maria Söderlund-Venermo
Kee hoon Sohn
Evgeni Sokurenko
Samantha Soldan
Peter Solomon
Greg Somerville
Gregory Sonnenberg
Justin Sonnenburg
Joseph Sorg
Tania Sorrell
Claudio Soto
Gerald Spaeth
Stefania Spanò
Tim Sparer
Paul Spearman
Sabine Specht
Deborah Spector
Stephen Spector
Brad Spellberg
Vanessa Sperandio
Christina Spiropoulou
Hergen Spits
Serena Spudich
Shiranee Sriskandan
Ashley St John
E. V. Stabb
Colin Stack
Peter Staeheli
Christopher Staiger
Henry Staines
Jason Stajich
Christina Stallings
Leonidas Stamatatos
Thomas Stamminger
Sarah Stanley
Vincent Starai
Brian Staskawicz
Michael Stassen
Bärbel Stecher
John Steel
Chad Steele
Olivia Steele-Mortimer
Thilo Stehle
Alexander Steinle
Torsten Steinmetzer
Steffen Stenger
Grant Stentiford
Robin Stephens
Ioannis Stergiopoulos
Lawrence Stern
Jeremy Sternberg
Paul Sternberg
Alasdair Steven
Mary Stevenson
Philip Stevenson
James Stewart
Adrie Steyn
Scott Stibitz
Benoit Stijlemans
Monique Stins
Mark Stinski
Patricia Stock
Paul Stoodley
Henrik Stotz
Jose Stoute
Jonathan Stoye
Michael Strand
Klaus Strebel
Hendrik Streeck
Daniel Streicker
Adrian Streit
Roland Strong
Richard Strugnell
Natalie Strynadka
Lynda Stuart
Gerald Stubbs
David Studholme
Jason Stumhofer
Chunlei Su
Xin-zhuan Su
Carlos Subauste
Kanta Subbarao
Sriram Subramaniam
Peter Sudbery
Christine Suetterlin
Bill Sugden
Christopher Sullivan

William Sullivan
Paul Sumby
Joseph Sun
Peter Sun
Ren Sun
Wenxian Sun
Shao-Cong Sun
Surachai Supattapone
Camille Sureau
Michael Surette
Witold K Surewicz
Mehul Suthar
Gerd Sutter
Fayyaz Sutterwala
Tetsuro Suzuki
Catharina Svanborg
Sankar Swaminathan
Michele Swanson
Michelle Swanson-Mungerson
Ronald Swanstrom
Gulam Syed
Yuri Sykulev
Michael Szostak
Makoto Takeda
Toru Takimoto
Frank Takken
Kawsar Talaat
Karel Talavera
Nicholas Talbot
Yunhao Tan
Hengli Tang
Wei-jen Tang
Herbert Tanowitz
Caroline Tapparel
Vera Tarakanova
Isabelle Tardieux
Rick Tarleton
David Tarpy
Ann Tate
Jeffery Taubenberger
Norbert Tautz
Jesse Taylor
John Taylor
Terrie Taylor
John Teijaro
Luis Teixeira
Alice Telesnitsky
Glenn Telling
Timothy Tellinghuisen
Lesly Temesvari
Italo Tempera
Benjamin tenOever
Ryohei Terauchi
Cox Terhorst
Scott Terhune
Vernon Tesh
Ken Teter
Guillaume Tetreau
Hervé Tettelin
Rita Tewari
Markus Thali
David Thanassi
Vaskar Thapa
Ulrich Theopold
Volker Thiel
Dennis Thiele
Robert Thimme
Paul Thomas
Bart Thomma
Hans Thordal-Christensen
David Thorley-Lawson
Stephen Thorne
Justin Thornton
Scott Tibbetts
Leann Tilley
Jens Tilsner
Rabindra Tirouvanziam
Peter Tobias
Niraj Tolia
Ana Tomás
Massimo Tommasino
Jan Tommassen
Keizo Tomonaga
Dan Tompkins
Stephen Tompkins
Qiong Tong
Tone Tonjum
Christopher Tonkin
Jordi Torrelles
Victor Torres
Domenico Tortorella
Zsolt Toth
Lhousseine Touqui
Paula Trakman
Paula Traktman
Ana Traven
Katharine Trenholme
Patrick Trieu-Cuot
Steven Triezenberg
Mirko Trilling
Ralph Tripp
Emily Troemel

Billy Tsai
Ching-Hsiu Tsai
Harley Tse
Renée M. Tsolis
Mizushima Tsunehiro
Bettina Tudzynski
Cagla Tukel
Nilgun Tumer
Terrence Tumpey
Elaine Tuomanen
B. Turgeon
Ted Turlings
Joseph Turner
Paul Turner
Stephen Turner
Rodney Tweten
Brett Tyler
Sukathida Ubol
Irina Udalova
Takashi Ueda
Glen Ulett
Buddy Ullman
Elisabetta Ullu
David Underhill
Leonie Unterholzner
Derya Unutmaz
Susan Uprichard
Jason Upton
Constantin Urban
Michael Urbaniak
Kevin Urdahl
Edward Usherwood
Mart Ustav
Jude Uzonna
Lisa Vaillancourt
Oswaldo Valdes-Lopez
Pedro Vale
Barbara Valent
Michael Valentino
Richard Vallee
Debbie van Baarle
Frank van de Veerdonk
Jan Van Den Abbeele
Guido Van den Ackerveken
Sjoerd van der Burg
Luc van der Laan
Tom van der Poll
Ando van der Velden
Nicole van der Wel
Jan Maarten van Dijl
Giel van Dooren
Linda van Dyk
Thomas Van Dyke
Peter van Endert
Peter van Esse
Luc Van Kaer
Jan van Kan
Lucien van Keulen
Frank van Kuppeveld
René van Lier
Rene van Lier
Carine Van Lint
Ronald van Rij
Jos van Strijp
Wesley Van Voorhis
Ger van Zandbergen
Russell Vance
Scott Vande Pol
Dennis vanEngelsdorp
Ramakrishna Vankayalapati
Steven Varga
Michael Vasil
Jiri Vavra
Andres Vazquez-Torres
Ronald Veazey
Jan-Willem Veening
Andre Veillette
Vittorio Venturi
Jeanmarie Verchot-Lubicz
Bruno Verhasselt
Subhash Verma
David Vermijlen
Gijs Versteeg
Viiva Vezys
Muriel Viaud
Cecile Viboud
Silvia Vidal
Ana Vigario
Marco Vignuzzi
Francois Villinger
Andreas Villunger
Boris Vinatzer
Amy Vincent
Mark Viney
Donald Vinh
Karen Visick
Jörg Vogel
Loy Volkman
Waldemar Vollmer
Thomas von Hahn
Andreas von Tiedemann
Frank Voncken

Christoph Vorburger
Frank-Jörg Vorhölter
M. Vos
Leslie Vosshall
Daniel Voth
Jovanka Voyich-Kane
Jatin Vyas
Alan W Decho
Simon Waddell
Joseph Wade
Stephen Waggoner
Matthew Waldor
Christopher M. Walker
David Walker
Graham Walker
Lary Walker
Suzanne Walker
Jacco Wallinga
Sarah Walmsley
Thierry Walzer
Gerhard Walzl
Cecile Wandersman
Jack Wands
Aiming Wang
Eddie Wang
Fu-Sheng Wang
Ming-Bo Wang
Robert Wang
Xiaofeng Wang
Xiaohong Wang
Zonghua Wang
Kasper Wang
E. Sally Ward
Gary Ward
Honorine Ward
Matthew Wargo
Digby Warner
Karen Wassarman
Andrew Waters
David Watkins
Paula Watnick
David Waugh
Michael Way
Friedemann Weber
Taiyun Wei
Christopher Weidenmaier
J. Brice Weinberg
Janis Weis
John Weis
Jeffrey Weiser
Brian Weiss
David Weiss
Louis Weiss
Robin Weiss
Susan Weiss
Winfried Weissenhorn
James Weisshaar
Matthew Weitzman
Matthew Welch
Sandra Weller
Dave Wemmer
Annemarie Wensing
David Wentworth
Joel Wertheim
Catherine Werts
Gail Wertz
Michael Wessels
Michelle West
Robert Wheeler
Sean Whelan
Stephen Whisson
Denise Whitby
Judith White
K. Andrew White
Michael White
Adrian Whitehouse
Marvin Whiteley
Malcolm Whiteway
Anna Whitfield
Steven Whitham
Jason Whitmire
Gary Whittaker
Ian Wickersham
Reed Wickner
Matthew Wiebe
Ursula Wiedermann
Mark Wiener
Emmanuel Wiertz
Chris Wiethoff
Brian Wigdahl
Stephen Wikel
Clayton Wiley
Gavin Wilkinson
Angela Wilks
Holger Wille
Lucas Willems
Sven Willger
Bryan Williams
David Williams
Kenneth Williams
Matthew Williams
Peter Williamson

Mark Wills
Angus Wilson
Joyce Wilson
Mark Wilson
Patrick Wilson
Paul Wingfield
Malcolm Winkler
Gary Winslow
Sebastian Winter
Roger Wise
Elizabeth Wohlfert
James Wohlschlegel
Thorsten Wolff
Matthew Wolfgang
Adrian Wolstenholme
Christiane Wolz
Elizabeth Wonderlich
Wilson Wong
Charles Wood
Craig Woodworth
Linda Wooldridge
Stefan Worgall
Floyd Wormley, Jr.
Daniel Wozniak
Jens Wrammert
David Wright
Gerard Wright
Jianguo Wu
Joseph Wu
Li Wu
Louisa Wu
Min Wu
Ting-ting Wu
Martin Wubben
Richard Wyatt
Michelle Wykes
David Wyles
Thomas Wynn
Mariana Xavier
Jun Xia
Yan Xiang
Gutian Xiao
Zhou Xing
Jin-Rong Xu
Chaoyang Xue
Hai-Hui Xue
Timothy Yahr
Masahiro Yamamoto
Yoshio Yamaoka
Takeshi Yamasaki
Masahiro Yamashita
Yohei Yamauchi
Seiya Yamayoshi
Yusuke Yanagi
Otto Yang
Soo-Jin Yang
George Yap
Neil Yarlett
Felix Yarovinsky
Andrew Yates
Maria Yazdanbakhsh
Ellen Yeh
Jonathan Yewdell
MinKyung Yi
Michael Yin
Dongwan Yoo
Hiroki Yoshida
Tamotsu Yoshimori
Dahui You
Carolyn Young
Howard Young
Kevin Young
Paul Young
Benjamin Youngblood
Je-Wook Yu
Xiao-Fang Yu
Peihua Yuan
Weiming Yuan
Yan Yuan
Andrew Yurochko
Jose Yuste
Bourne Yves
Eliora Z Ron
Mark Zabel
Olga Zaborina
Allan Zajac
Dirk Zajonc
Xingxing Zang
Agustín Zapata
Michael Zasloff
Fidel Zavala
Teresa Zelante
Kornelius Zeth
Jian Zhang
Li Zhang
Shuqun Zhang
Xuming Zhang
Zhang Zhang
Zhi-Ming Zheng
Guangming Zhong
Bin Zhou
Daoguo Zhou

Hong Zhou
Jian-Min Zhou
Fanxiu Zhu
Jun Zhu
Yongqun Zhu
John Ziebuhr
Wilma Ziebuhr
Joseph Ziegelbauer
Gert Zimmer
Richard Zimmermann
Cyril Zipfel
Simona Zompi
Wen-Quan Zou
Richard Zuerner
Jian Zuo
Chiara Zurzolo
Mark Zwart
Michael Zwick
Arturo Zychlinsky
